# MiteFinderII: a novel tool to identify miniature inverted-repeat transposable elements hidden in eukaryotic genomes

**DOI:** 10.1186/s12920-018-0418-y

**Published:** 2018-11-20

**Authors:** Jialu Hu, Yan Zheng, Xuequn Shang

**Affiliations:** 1 School of Computer Science, Northwestern Polytechnical University, West Youyi Road 127, Xi’an, 710072 China; 2 Centre of Multidisciplinary Convergence Computing, School of Computer Science, Northwestern Polytechnical University, Dong Xiang Road 1, Xi’an, 710129 China

**Keywords:** Transposable element, K-mer index, Genomic analysis, Terminal inverted repeat, Target site duplication

## Abstract

**Background:**

Miniature inverted-repeat transposable element (MITE) is a type of class II non-autonomous transposable element playing a crucial role in the process of evolution in biology. There is an urgent need to develop bioinformatics tools to effectively identify MITEs on a whole genome-wide scale. However, most of currently existing tools suffer from low ability to deal with large eukaryotic genomes.

**Methods:**

In this paper, we proposed a novel tool MiteFinderII, which was adapted from our previous algorithm MiteFinder, to efficiently detect MITEs from genomics sequences. It has six major steps: (1) build K-mer Index and search for inverted repeats; (2) filtration of inverted repeats with low complexity; (3) merger of inverted repeats; (4) filtration of candidates with low score; (5) selection of final MITE sequences; (6) selection of representative sequences.

**Results:**

To test the performance, MiteFinderII and three other existing algorithms were applied to identify MITEs on the whole genome of oryza sativa. Results suggest that MiteFinderII outperforms existing popular tools in terms of both specificity and recall. Additionally, it is much faster and more memory-efficient than other tools in the detection.

**Conclusion:**

MiteFinderII is an accurate and effective tool to detect MITEs hidden in eukaryotic genomes. The source code is freely accessible at the website: https://github.com/screamer/miteFinder.

## Background

**Fig. 1:**
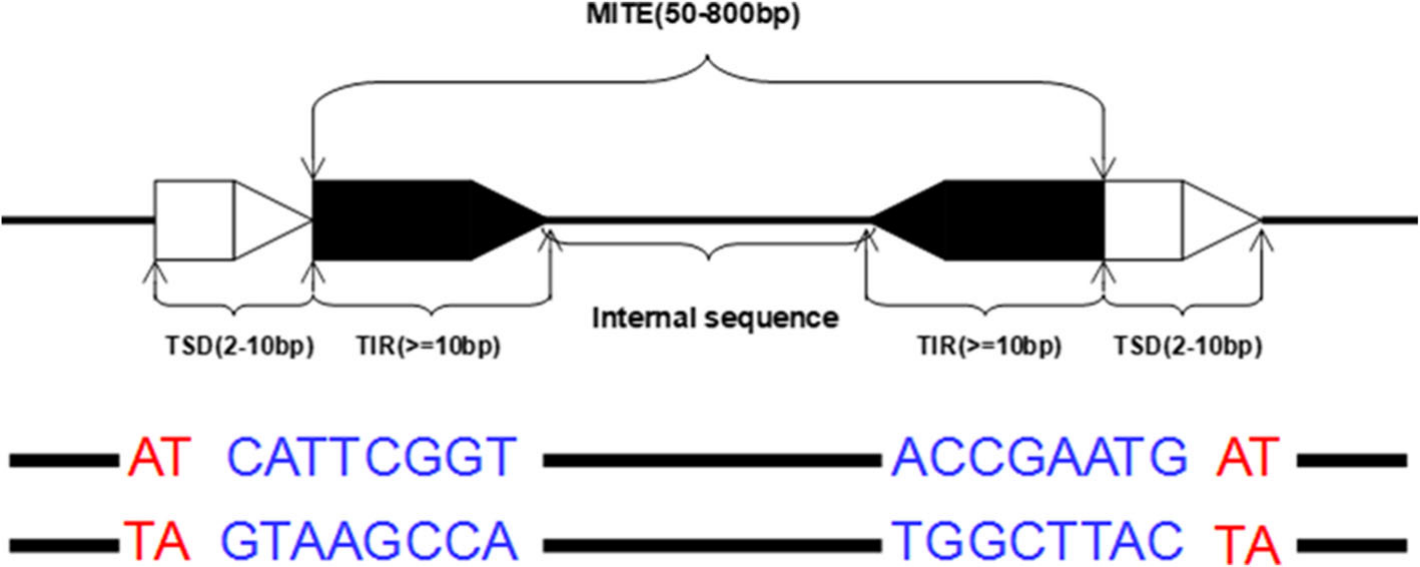
The typical structure of a miniature inverted repeat transposable element. The length of MITE is between 50–800bp, and a complete MITE contains a pair of terminal inverted repeat(TIR) and a internal sequence. MITEs usually are flanked by a target site duplication(TSD). The following example sequence is a typical MITE sequence, the blue part is the TIR of MITE and the red part is the TSD

Transposable elements (TEs) are present in many plants and animals, which make up of a large proportion of the genome. For example, 85% of the maize genome is made up of TEs [1], as is 46% of the human genome [2]. Transposable elements have contributed to evolution by causing gene variants and altering genomic structures and regulation of individual genes. It suggests that TEs are important in genome function and evolution. There are two major categories of TEs according to molecules involved in transposition: Class II TEs (also called retrotransposons) move through RNA intermediates, which can be described as copy and paste; Class II TEs (known as DNA transposons) encode the protein transposase, which can be described as cut and paste. Not all DNA transposons transpose through the cut-and-paste mechanism. TEs are also classified as autonomous and non-autonomous TEs based on whether they can move by themselves.Generally, non-autonomous TEs require another TE to move. Miniatures inverted repeat transposable element (MITE) is a special type of non-autonomous DNA transposons, which has a special structural feature and higher copy numbers in eukaryotic genomes. As shown in Fig.1, MITE is a DNA sequences with about 50–800 bp in genome, which contains short conserved terminal inverted repeats (TIR, >= 10bp) and an internal sequence. The whole MITE is flanked by a pair of target site duplication (TSD, about 2–10 bp in length)[3]. Because the MITEs are non-autonomous TEs, MITEs do not encode the proteins and have no coding potential for their transposition. Theoretically, MITEs should have perfect inverted repeats. Actually, there are a large number of MITEs without perfect inverted repeat (inverted repeats with some mismatches). In Fig.1, the following sequence is a MITE sequence with perfect inverted repeats.

MITEs are associated with gene regulation in angiosperms. They play important roles in genome evolution. The movement of MITEs in genes may alter their structure and function and play a significant role in the evolution of organisms. For example, a big MITE family named stowaway in potato was found to cause phenotypic diversity of skin color by altering the structure of related genes [4].

Therefore, there is an urgent demand for the development of bioinformatics tools to accurately and efficiently detect MITEs in whole genomes. It would help us get a better understanding of the gene regulatory mechanism in genome-wide association studies [5–8].

To identify TE on a genome scale, three existing tools have been developed, including MITE-Hunter [9], MITE Digger [10] and detectMITE [3]. MITE-Hunter firstly finds all possible candidates based on the TIR-like structure, and then filters false positive ones by using pairwise sequence alignment and multiple sequence alignment, generates exemplars and groups all MITEs into families. MITE Digger searches for MITEs using redundant computing and then reduces the redundancy by computing a representative of the family. The latest developed tool detectMITE employs a numeric calculation approach to replace string matching algorithm in the MITE detection, adopts the Lempel-Ziv complexity algorithm to filter out candidates with low complexity and utilizes CD-Hit to cluster them into different families. However, both MITE-Hunter [11] and MITE Digger save the computation complexity by sacrificing sensitivity and precision [12]. The algorithm of detectMITE is not only time-consuming, but it requires large computational resources. It is hard to run detectMITE on large genomes with a machine with moderate memory.

Due to these weak points of current MITE detection tools, it is necessary to develop a more accurate and effective tool to study the MITEs in a genome-wide scale. However, there are two basic challenges: (1) identification of TIR-like structure from a whole genome: (2) filtration of false positive candidates.

To solve these problems, we proposed a novel computational tool MiteFinder [13], which can accurately, comprehensively and efficiently detect MITEs in a whole genome. Meanwhile, it is more memory-efficient and much faster than all existing tools by building k-mer index for genomic fragments. MiteFinderII is extended from MiteFinder. Compared with MiteFinder, MiteFinderII adds new function to cluster MITE sequences into different MITE families and it is easy and simple to be executed by non-professional users.

## Methods

**Fig. 2:**
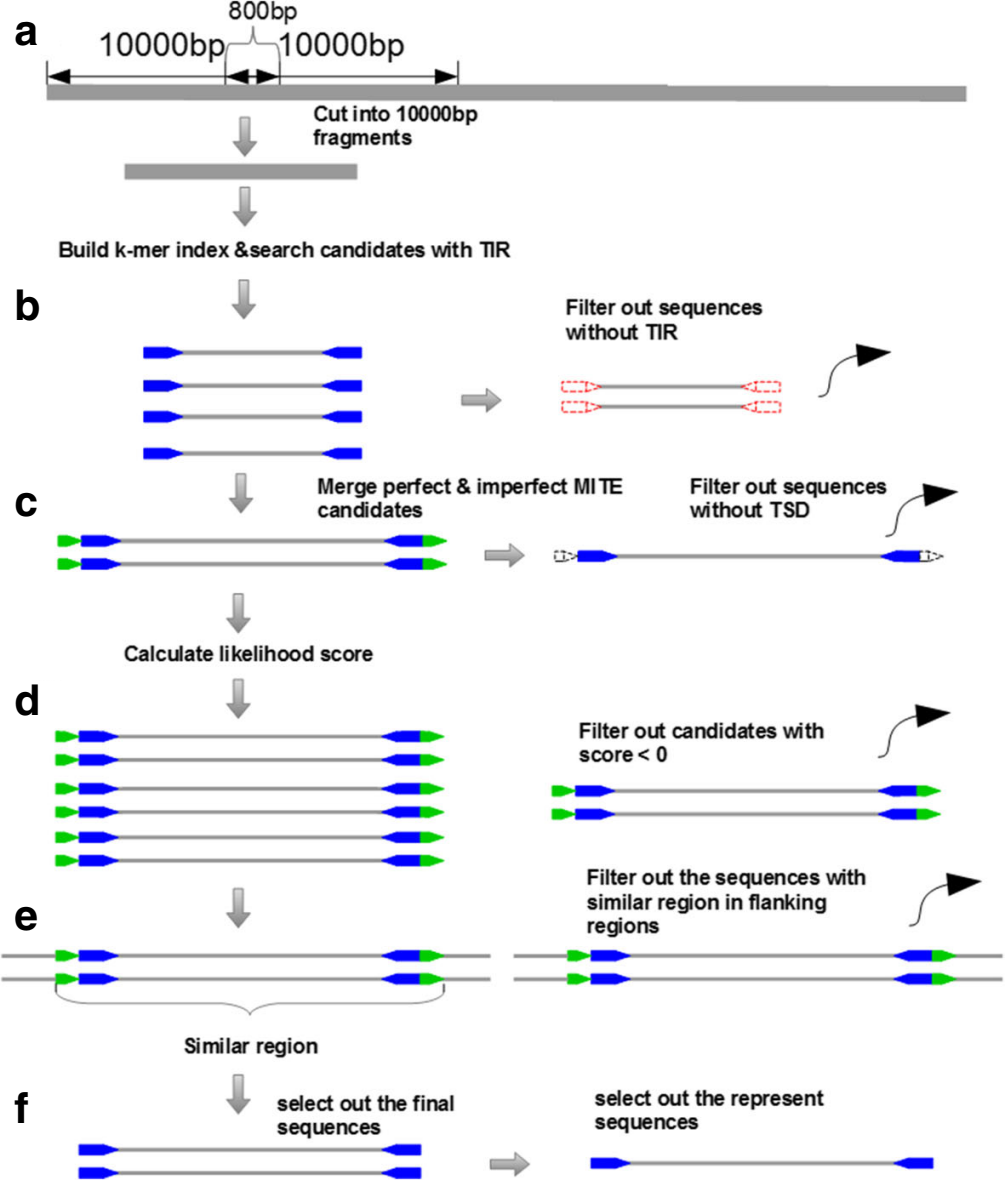
The core algorithm of MiteFinderII in the MITE detection. **a** A whole genome was cut into many pieces of sequence fragments and the build of k-mer index for each fragment. **b** Search for sequences seeds by using k-mer index. Each seed requires at least 10bp inverted repeats (TIR structure); **c** Extend these seeds to a complete MITE structure by merging smaller ones, including TSD structure; **d** Calculate the likelihood score for each MITE candidate using log-ratio model, filter away these candidates with low score; **e** filter these candidates with similar sequences in the flanking regions. **f** Select out the represent sequences of MITE families

In order to make improvements for existing tools, we have developed a new program in C++ language, MiteFinderII, which can be used to detect both perfect inverted repeats and imperfect inverted repeats utilizing the string matching approach [14]. As non-autonomous DNA transposons, the structure of MITE is characterized by the terminal inverted repeats. All existing tools consumed most of the time searching for all possible terminal inverted repeats [15]. To speed up the computation in this stage, we employ a hash function to build index for each k-mer in the sequence fragments. Compared to our previous tool, MiteFinderII can set up the parameter in linux command, which is more convenient to use. The input data of program is genome sequences in the FASTA format. The genome of rice contains a large number of transposable elements, and rice is a model plant for genome science of grasses since its genome sequence has been completely determined. MITEs have the highest copy number among transposable elements in rice [16], which constitute approximately one-third of the genome sequence [17]. The whole genome sequence of rice can be downloaded on internet freely, so the genome of rice is selected as our test data (MSU Rice Genome Annotation Project Release 6.1, 369 Mega Byte). The test data is the genome sequences in the FASTA format, which has been downloaded from the NCBI website. As shown in Fig.2, the core algorithm of MiteFinderII consists of five major steps. The detailed description of each step is introduced in the following sections.

### Build K-mer index and search inverted repeats

Firstly, we attempt to detect all possible inverted repeats. In the FASTA format of genome sequences, the first line is headed by ‘ >’, followed by its literal description. The chromosome data starts from the second line. It ends until it reaches next chromosome. In MiteFinderII, every chromosome will be traversed to get the size of every chromosome and then every chromosome will be stored in an optimum amount of memory to be more memory-efficient. Memory will be released automatically at the end of the program. For an input genome, all sequence fragments that have a TIR pair (>= 10 bp in length) and a TSD pair (2–10bp) will be identified. We identify inverted repeats by TIR pair detection in program. First, each chromosome sequences will be divided into multiple sequence fragments with same length (default 10,000 bp). There is a common sequence (800 bp, the maximum length of MITE) between the adjacent fragments to ensure that all inverted repeats are identified. Secondly, we divide the sequence fragment into multiple adjacent fragments with length is 10 bp, all inverted repeats (the length of TIR >= 10 bp, so the initial length of TIR in inverted repeats is 10 bp) will be stored in an unordered_map (as k-mer index). The key of unordered_map is used to storage the sequence of inverted repeats and the value is used to store the position of inverted repeats with same sequence (key is a string and value is a vector of integer). We can retrieve the position of inverted repeats in the unordered_map. The program utilizes unordered_map since it can fast retrieve a certain sequence in detection [18]. There is a pair of TIR and an internal sequence in a MITE, so we create a function in program to obtain the inverted repeat sequence of inverted repeat. We detect all pairs of TIR that can match each other. The pairs of TIR with length between 50–800 bp will be retained. The rest pairs of TIR will be used as seeds of MITE candidates in next step.

### Filtration of inverted repeats with low complexity

**Fig. 3:**
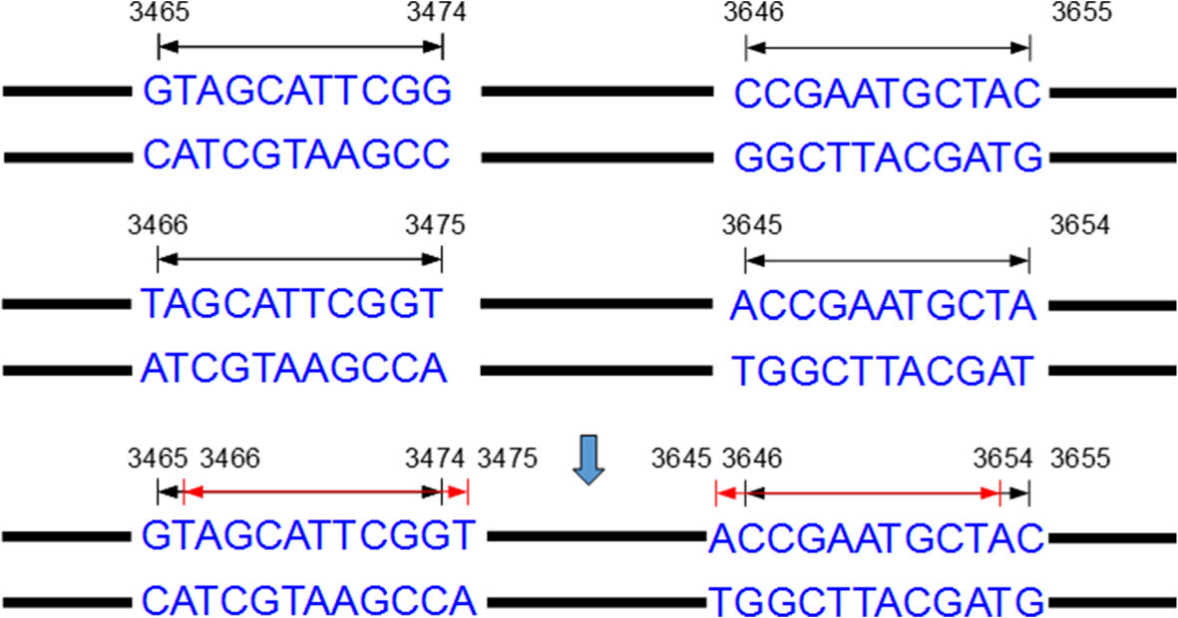
Two adjacent perfect inverted repeats with the length of 10bp were merged into a bigger one with the length of 11bp

All inverted repeats will be stored in a list including the starting position and ending position of each inverted repeats. These inverted repeats with low complexity should be filtered out, since they are less likely to be in MITE families. First, there are two TIR in an inverted repeat. Each putative TIR that meets one of the following criteria was filtrated as low complexity cases to improve the efficiency and accuracy: (1) it contains >= 8bp homopolymer or dinucleotide in TIR; (2) it contains < 20% G/C or A/T content. All seeds of MITE candidates will be identified after all inverted repeats is determined. Next, the adjacent inverted repeats must be merged because the inverted repeats belong to the same MITE candidate. As shown in Fig.3, an inverted repeat with a TIR of 11 bp in length will be divided into two inverted repeats stored in a list. In this step, our work is to merge inverted repeats as shown in Fig.3. The merged inverted repeats will be selected in next step as MITE candidates. However, most inverted repeats are incomplete, so some inverted repeats without adjacent position should be merged.

### Merger of inverted repeats

**Fig. 4:**
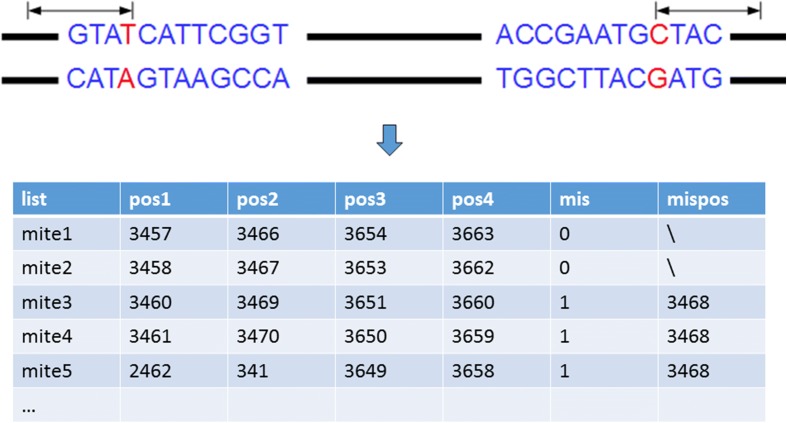
An example of imperfect MITE candidates, which has one mismatch base. In this example, the mismatch position in the left hand of TIR is 3468. And all these short candidates in the table were merged into a longer imperfect MITE

In Fig.3, it shows how the adjacent inverted repeats should be merged. For perfect inverted repeats, we merge them by retaining one of them and altering the position information. However, not all inverted repeats can match perfectly, and the imperfect inverted repeats must be considered. Imperfect inverted repeats are abundant in MITEs. Therefore, inverted repeats with some unmatched base pair (default = 1) are also considered to be MITE candidates. We should retain the imperfect inverted repeats in the detection of TIR pairs. So we modified the function of extract_seed_from_map and storage the imperfect inverted repeats in the list. The function will find the all TIR candidates that have only one base differed from the perfect inverted repeats and get all imperfect inverted repeats. The function only obtains the imperfect inverted repeats that mismatch position does not appear in the start and end. The function of merge MITE also is modified to deal with the problem that a MITE candidate missing when a complete imperfect inverted repeat is merged. We add two parameters named ‘mis’ and ‘mispos’ in the function. The ‘mis’ is used to record the MITE is perfect or imperfect and ‘mispos’ is used to record the position of mismatch. An imperfect inverted repeat is shown in Fig.4, the red base is the position of mismatch. The position of the front red base is 3468 and the back is 3652. The MITE candidates will be stored in list just like Fig.4. The MITE candidates contain the mismatch base will store the position of the mismatch base and the parameter of ‘mis’ is 1. When an imperfect MITE and a perfect MITE merge, the difference between positions of TIR is 2 is also obtained and the ‘mis’ is recorded by 1 and ‘mispos’ is recorded by the position of mismatch. When two perfect MITEs merges, we utilize same method to merge the MITE candidates in the last step. The merger of two imperfect MITEs will be filtration as the false positive case. After the merger of inverted repeats, MITE candidates that length of TSD is not between 2–10 bp or if the length of TSD is 2 and TSD is not ‘TA’ were filtrated out as false positive cases.

### Filtration of candidates with low score

From the above steps, we obtain complex MITE candidates which have TIR-like structure and TSD structures. To improve the precision, we create a scoring formula to filtrate the false positive cases in the rest MITE candidates. We create a model named MITE model, which contains more than 30,000 MITE sequences. A null model contains more than 160,000 sequences. which include both non-MITE sequences. The positive MITE sequences are the sequences that have high similarity with the MITEs have been found in the Repbase database. The false positive MITE sequences are the inverted repeat sequences found in genome with complete MITE structure but have low similarity with the MITE in Repbase. We deal the sequences as follows. We divide the all MITE sequences into the fragments that length is 6 bp. We calculate the sum of every fragment that appears in the positive MITE sequences and false positive MITE sequences. For the MITE candidates found in the third step, we divide every sequence into fragments with the same length. For every given fragments S that length is 6 bp, the score of S is:

$$F(s)=\log_{2} \frac{Pr(S/M)}{Pr(S/N)} $$

S/M is the probability of S appears in M (M is the positive MITE sequences data set), S/N is the probability of S appears in N (N is the false positive MITE sequences data set). We assume that the longer of the sequence, the more times of the different fragments appears in the sequence. The effects of the length must be considered, so the score of sequence must be divided by length to eliminate effects. The score of sequence that length is n is:

$$Score=\sum\limits_{i=1}^{N-5} F(i)/(n-5) $$

We get all scores of the MITE candidates from the above formula. In MiteFinderII, the parameter of scores can be set up by user. After several tests, we choose 0 as the default criteria, the MITE candidates that score greater than 0 were retain as the true positive MITEs, and the rest MITE candidates were filtrated.

### Selection of final MITE sequences

In the previous steps, the MITE candidates have been preliminary screening. The further screening is necessary to improve the accuracy of program. MiteFinderII clusters MITE candidates with a pair of flanking sequence (length is 60 bp) into the distinctive families based on their sequence similarity by all-by-all BLASTN comparison (default e-value =1e-10). When a MITE is transposed in the genome, it is less likely that its flanking sequences are transposed together, so we discard the MITEs that share similarity in their flanking sequence. From the results of BLASTN, each putative MITE that meets all of the following criteria was retained as true positive case: 1. pident value >80*%*, 2. those MITE candidates that share sequence similarity within but not in their flanking regions. The remaining MITE sequences are the sequences for MITE families.

### Selection of represent sequences

Compared with MiteFinder, MITE sequences we got in the last step are the final MITE sequences in rice. Unlike the traditional low copy non-autonomous TEs, the MITEs amplify rapidly from one or few elements to high copy numbers. Hence, similar sequences should be clustered into a MITE family. We compared MITE sequences (without flanking sequences) identified in the last step with each other by BLASTN. In the previous test, we found that different MITE sequences have high similarity. We choose 1e-100 as the criteria for clustering. We use the results of BLASTN to build a network. These nodes clustered in a same group consist of a MITE family. For each cluster, only these group with more than three members were retained as valid MITE families. The MITE sequences with highest degree in each MITE families were selected as the representive MITE sequences. Finally, 11,239 MITE families in rice genomes are identified.

## Results

To test the accuracy and efficiency, we performed other three existing tools detectMITE, MITE Digger and MITE-Hunter to detect MITEs from the oryza sativa genome. The MiteFinderII, detectMITE and MITE-Hunter performed in Ubuntu system with one core. Since the Linux version of MITE Digger is not available, we performed MITE Digger in windows with one core.

### The efficiency of MiteFinderII

**Fig. 5:**
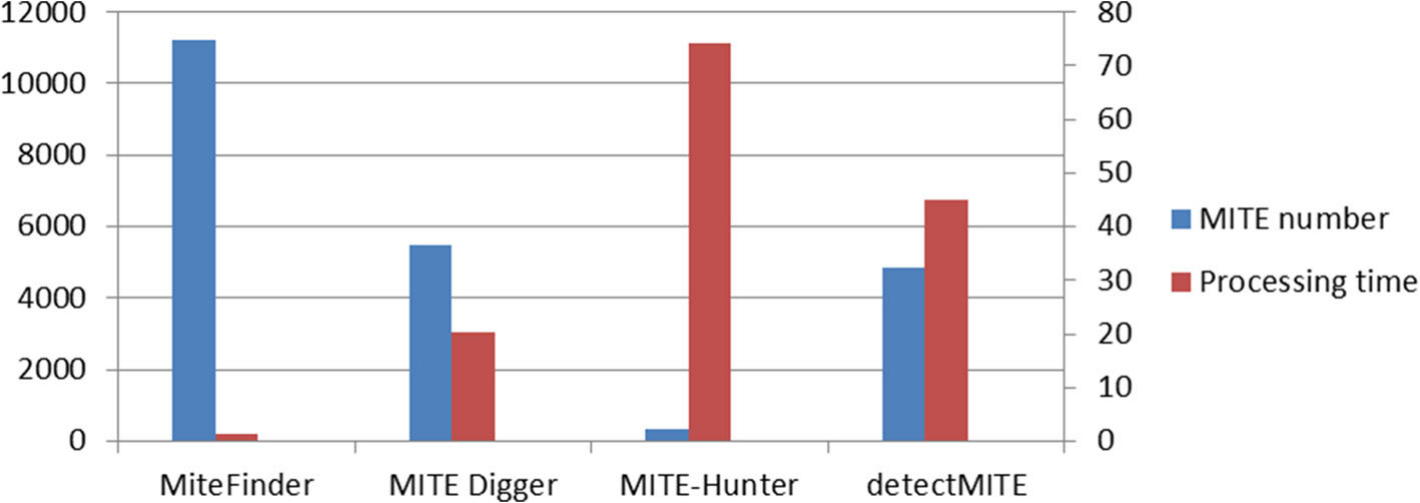
MITE number and processing time. MITE number is the total number of MITE sequences that each tool can identify on the rice genome and processing time is the total time of each tool running on the rice genome with 1 CPU core

As shown in Fig.5, MiteFinderII spent only 1 h and 20 min to detect MITEs from the whole genome of rice and 11,239 MITE families were identified (include 26,704 MITE sequences). In contrast, detectMITE took 44.94 h and found 4838 MITE families, MITE-Hunter took more than 70 h and found 333 MITE families with the length between 50 and 800bp (parameter: max group is 1 and the number of CPU is 1). MITE Digger took 20 h and 15 min to detect 5499 MITE sequences that have a complete TIR and TSD structure (50–800 bp) in the whole genome. Although MITE Digger is running on different system, it is obvious that MiteFinderII is more efficient than other tools.

The sequence comparison is time-consuming by using BLASTN. It takes only 1361 s to detect MITE candidates, excluding the clustering step on networks. MiteFinderII was also performed on other datasets. It costs 2273 s and 6751 s in the detection of MITEs on Sorghum (684 Mega Byte) and zea mays (2058 Mega Byte), respectively. MiteFinderII takes about 1 h to detect MITE candidates on a genome of one Giga Bytes.

### The distribution of superfamily of MITEs

**Fig. 6:**
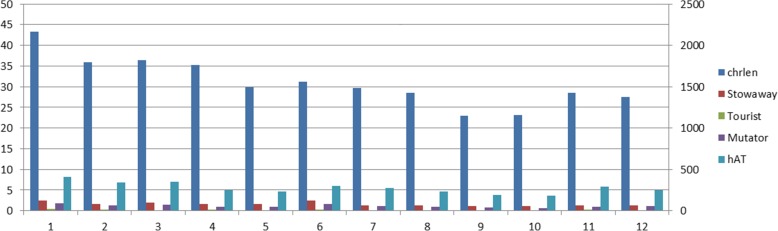
The distribution of four MITE family sequences on the 12 rice chromosomes. The left ordinate is the size of chromosomes. The right ordinate is the number of different super families.The abscissa is the number of chromosomes. The length of the 12 rice chromosomes is 43.27M, 35.93M, 36.41M, 35.28M, 29.90M, 31.25M, 29.70M, 28.44M, 23.01M, 23.14M, 28.51M and 27.50M, respectively

There are two major superfamilies of MITEs, named stowaway and tourist. Stowaway is a superfamily of MITEs with ‘TA’ as the TSD, which is widespread and abundant in plant genomes. Tourist is a superfamily of MITEs with ‘TAA’ as the TSD. There are some other superfamilies such as hAT (5, 6, 8 bp TSDs) and Mutator (9, 10 bp TSDs) [19]. In plant genomes, these families have hundreds of copies and can change the structure of genes. The transposition is strongly related to the diversity and evolution of genes. We classify MITE sequences by TSD into different superfamilies and study the distribution of MITEs in rice genome. There are 963 Stowaway MITE sequences, 140 Tourist MITE sequences, 690 Mutator MITE sequences and 3314 hAT MITE sequences in the result of MiteFinderII. The distribution of all MITE sequences are shown in Fig.6.

As shown in Fig.6, there doesn’t exist an obvious linear correlation between super families and chromosomal length. The Stowaway sequences are abundantly distributed in chromosome 6 and chromosome 1. Tourist sequences in chromosome 12 are less than these in chromosome 9, 10. There are only 7 tourist sequences in chromosome 12.

### The accuracy of MiteFinderII

To evaluate the performance of MiteFinderII, we performed MiteFinderII and other existing tools on datasets from the Repbase database [20]. Repbase is a comprehensive repeat database that contains both transposon elements and other repeats. It has been widely utilized in genome annotation. According to the characteristics of MITE, we extracted out all complex non-autonomous DNA transposable elements of rice. We found 1437 complex non-autonomous DNA transposable elements in oryza sativa genome, 547 of which have a length of 50–800 bp. The sequences extracted from Repbase are the complex non-autonomous DNA transposable elements of rice. MITEs sequences in Repbase have been updated regularly. We can always find many new MITE sequences after it was updated every time. Compared to MiteFinder, MiteFinderII has a better performance in terms of recall. We also compared the results of MiteFinderII with that of three other algorithms, MITE Digger, detectMITE and MITE-Hunter using BLASTN (e-value =1e-10 as a threshold). The results are shown in Table 1. The match number is the number of MITE sequences that can match a similar sequence in Repbase. The Repbase number is the number of sequences of Repbase that can match a similar sequence in the results of each tool.

**Table 1:** The results of four algorithms in the detection of MITEs on the rice genomes

Tools	MITE number	Match number	Repbase number
detectMTIE	4838	1461	213
MITE Digger	5499	1847	194
MITE-Hunter	333	109	112
MiteFinderII	11,239	2631	287

As shown in Table 1, detectMite found 4838 MITE families, 1461 of which can match with 213 reference MITE sequences in Repbase. The precision is 33.59% and the true positive rate is 35.47%. MITE Digger found 5499 MITEs, 1847 of which match with 194 MITEs in Repbase. The precision is 30.20% and the true positive rate is 38.90%. The output files of MITE-Hunter include multiple alignment files and consensus TE sequences grouped into families. MITE-Hunter detected 303 MITE families. The algorithm of detectMITE found 109 MITE families, The precision is 32.73% and the true positive rate is 20.48%. MiteFinderII detected 11,239 perfect MITEs, 2631 of which match with 287 MITEs of Repbase. The precision is 23.41% and the true positive rate is 52.47%. To evaluate the performance, we used F-measure as a standard measure to evaluate the performance of MiteFinderII and other existing tools. F-measure is a common evaluation standard in information retrieval [21], which can be written in the following formula,

$$F_{\beta}=\frac{\left(\beta^{2}+1\right)precision*recall}{\beta^{2}(precision+recall)}. $$

F1-score is commonly used, which is *β*=1.

Here, the reference sequences of Repbase are non-autonomous transposable elements of rice genome. As shown in Table 2, MiteFinderII and detectMITE have the highest F-score (0.42), followed by MITE Digger. MiteFinderII has the best performance in recall, while detectMITE has the best performance in terms of precision. MITE-Hunter has a good performance in precision, but it has the smallest score in recall (Recall =TP/TP+FN and Precision =TP/TP+FP).

**Table 2:** The performance of four algorithms on recall, precision and F1-measure

Tools	Recall	Precision	F-measure
detectMTIE	35.47*%*	33.59*%*	0.35
MITE Digger	38.90*%*	30.20*%*	0.37
MITE-Hunter	20.48*%*	32.73*%*	0.22
MiteFinderII	52.47*%*	23.41*%*	0.42

## Discussion

MITE-Hunter, MITE Digger are notable existing tools, which can detect the ubiquitous MITEs hidden in eukaryotic genomes. Most tools have good performance in MITE detection due to the tir-like structure of MITEs. However, it is still a challenge to effectively detect MITEs in a genome-wide scale. The MITE-Hunter and MITE Digger that using both de novo and structure-based approaches can apparently improve the detect accuracy. However, some MITEs hidden in genome will be missed by MITE Hunter and MITE Digger. Comparing to existing tools, detectMITE has the best precision and F-measure, but it is memory-inefficient. It’s hard to run the program on a machine with moderate computational resources. Compared with detectMITE, MiteFinderII is fast and memory-efficient. It takes the shortest time and only 400M internal memory for the rice genome. Compared with detectMITE, MITE-Hunter and MITE Digger, MiteFinderII detect more MITEs and MITE sequences in Repbase. Compared with MITE-Hunter and MITE Digger, MiteFinderII has the best true positive rate. From the analyses, it is obvious that MiteFinderII outperforms other tools in terms of accuracy and efficiency.

## Conclusions

An efficient detection of MITEs from eukaryotic genomes is a crucial step for the understanding of gene mutation and regulation. Here, we introduce a novel algorithm, MiteFinderII, which can fast, accurately and comprehensively detect MITEs in whole genomes of eukaryotes. Hash functions were employed to build k-mer indexes for genomic fragments, which can speedup the retrieval of terminal inverted repeats using string matching approaches. A new log-ratio scoring model was designed to calculate the likelihood score of MITE candidates, which enables us to improve the accuracy of MITE detection. We performed MiteFinderII and all other existing tools on the same data of oryza sativa genome. The results show that MiteFinderII is more memory-efficient and much faster than all other existing tools. It is two orders of magnitude faster than detectMITE, which is the latest tool developed for MITE detection. Meanwhile, it can identify the most comprehensive MITEs in the rice genome with the best F-score. In addition, we carried out genome-wide analyses for the distribution of MITE families in different chromosomes. The results indicates that MITE sequences are widely distributed in the genome of rice and play important roles in the gene expression. We also performed MiteFinderII in other plant genomes. MiteFinderII provides many improvements to currently existing tools in the detection of MITEs, which would greatly benefit the research community working on the genome-wide association studies and function annotations.

## Abbreviations

MITE: Miniature inverted-repeat transposable element
TE: Transposable element
TIR: Terminal inverted repeats
TSD: Target site duplication
